# Investigation of a Novel Common Subexpression Elimination Method for Low Power and Area Efficient DCT Architecture

**DOI:** 10.1155/2014/620868

**Published:** 2014-07-16

**Authors:** M. F. Siddiqui, A. W. Reza, J. Kanesan, H. Ramiah

**Affiliations:** Department of Electrical Engineering, Faculty of Engineering, University of Malaya, 50603 Kuala Lumpur, Malaysia

## Abstract

A wide interest has been observed to find a low power and area efficient hardware design of discrete cosine transform (DCT) algorithm. This research work proposed a novel Common Subexpression Elimination (CSE) based pipelined architecture for DCT, aimed at reproducing the cost metrics of power and area while maintaining high speed and accuracy in DCT applications. The proposed design combines the techniques of Canonical Signed Digit (CSD) representation and CSE to implement the multiplier-less method for fixed constant multiplication of DCT coefficients. Furthermore, symmetry in the DCT coefficient matrix is used with CSE to further decrease the number of arithmetic operations. This architecture needs a single-port memory to feed the inputs instead of multiport memory, which leads to reduction of the hardware cost and area. From the analysis of experimental results and performance comparisons, it is observed that the proposed scheme uses minimum logic utilizing mere 340 slices and 22 adders. Moreover, this design meets the real time constraints of different video/image coders and peak-signal-to-noise-ratio (PSNR) requirements. Furthermore, the proposed technique has significant advantages over recent well-known methods along with accuracy in terms of power reduction, silicon area usage, and maximum operating frequency by 41%, 15%, and 15%, respectively.

## 1. Introduction

In the modern era, digital image processing has become widely used in electronic devices. A plethora of different multimedia applications spread rapidly, such as camcorders, cameras, video conferencing on mobile phones, online video streaming, video surveillance, patient monitoring systems, and high definition television (HDTV). These applications require a large amount of data to represent the digital images, resulting in large memory and transmission costs. Modern compression techniques play an important role to reduce the high storage and transmission cost. Image processing techniques have become more significant for various multimedia applications in embedded systems. Speed, power consumption, hardware area, resource usage, and throughput are the main criteria to be concerned in the development of image processing algorithm architectures. Especially, in portable systems, the key features are low power and low area with speed [1–6]. Thus, it has been the field of interest for the researchers.

Discrete cosine transform (DCT) is widely used in the majority of the international video/image standard coders [7]. In the recently published work, various high throughput DCT architectures have been designed to meet the requirement of real time applications [8–15]. DCT is one of the compute intensive parts in various image/video coding standards, such as JPEG (Jointed Photographic Practiced Group), H.261, H.263, and H.264/MPEG (Motion Pictures Practiced Group) [16, 17]. DCT transforms a signal or image from the spatial domain to the frequency domain. In emerging multimedia applications, DCT is widely used in portable systems, as they have limited CPU computing ability. Hence, it requires efficient hardware which consumes low power and low area and also satisfies the throughput criteria of the coder.

The DCT algorithm has excessive numbers of multiplication and addition operations. Different complex algorithms and architectures are designed for DCT implementation in the past. Some of them use complex flow graphs, butterflies structures, and systolic architectures to achieve higher throughput. Distributed Arithmetic (DA) based designs are also proposed for DCT [18, 19]. ROM-based DA architecture is proposed to reduce the area [9, 12]. Shams et al. [11] introduce New Distributed Arithmetic (NEDA) to implement DCT by using adder-based butterfly matrix. This new approach [11] utilized 35 adders and 8 shift-addition elements instead of ROM. Coordinate Rotation Digital Computer (CORDIC) based architectures are also one of the well-known DCT implementation schemes [20, 21]. But still there is a further scope for researchers to design an architecture which is a combination of some efficient techniques of DCT algorithms as well as optimization in hardware. Recent implementations focused on area and power to a considerable extent, but none of them seems to achieve the minimum possible area with low power. Therefore, the goal is to achieve less complex, efficient resource usage (minimum number of adders used) and low power system with high throughput.

In this paper, a novel architecture is proposed for DCT computation. It is based on the Canonical Signed Digit (CSD) encoding and use of Common Subexpression Elimination (CSE) technique. The efficient use of the CSE, not only in CSD encoding, but also in intermediate DCT coefficients, is introduced to compute the DCT results. Due to this approach, multiple identical subexpressions are needed to compute only once, which reduces the resources usage because of sharing the subexpressions. As a result, the total number of adders/subtractors required to compute DCT is reduced.

The rest of the paper is organized as follows.  Section 2 presents the materials and methods. Brief overview of DCT and recent published works are explained. The proposed system is also described in this section. Experimental results and comparisons are discussed in Section 3. Finally, conclusions are drawn in Section 4.

## 2. Materials and Methods

The DCT has become important and useful in various signal processing applications, especially speech and image compression. 1D-DCT for *N* points can be mathematically defined as

(1) F(k)=C(k)∑x=0N−1f(x)cos⁡(kπ(2x+1)2N),k=0,1,…,N−1,

where

(2) C(k)={1N,k=0,2N,k≠0,k=0,1,…,N−1.

The DCT algorithm is computationally intensive by nature. DCT computation has an excessive number of multiplications and additions operations. Therefore, according to the definition of DCT, algorithm as in (1) required *N* ^2^ multiplications and *N*(*N* − 1) additions. That means 4096 multiplications and 4032 additions are required for computing 8 × 8 2D-DCT.

Most of the high speed and real time multimedia applications need fast DCT algorithms and architectures. To increase the speed and overcome the extensive arithmetic operations of DCT computation, many fast DCT algorithms are proposed. There are many generalized DCT algorithms, such as Chen et al. [22], Lee [23], and Loeffler et al. [24] algorithms. Also, several recent literatures provide some evidences of algorithmic specific architectures, like DA [18], NEDA [11], CORDIC [20, 21], systolic architectures [12], and many more. Some of the major successful developments of DCT algorithms and different well-known implementations are briefly described in the following subsections.

### 2.1. Fast DCT Algorithm

Several research works based on fast DCT algorithms are reported in the past. All of them use the symmetry of the cosine function to reduce the number of multipliers.  Table 1 illustrates the number of arithmetic operations required in some of the most successful algorithms of fast DCT. In [24], the authors presented a fast DCT algorithm, which realizes the fast DCT with a minimum number of arithmetic operations. It required only 11 multiplications and 29 additions for computing 8-point DCT, which is the theoretical lower bound on the number of multiplications. Rotators (cosine/sine butterflies matrices) were also used in this design. This algorithm has 4 stages and each has to be executed in series and cannot be computed in parallel due to data dependencies. In stage 2, even coefficients and odd coefficients are separated by the algorithm. This algorithm requires a uniform scaling factor of $\sqrt{2}/4$ at the end of each output value to obtain the original 1D-DCT. However, the scaling factor is included in both DCT and IDCT so there will be no effect on the result of compression or in any other applications [25, 26].

### 2.2. Distributed Arithmetic Based DCT

DA is an efficient implementation for computing the inner partial product between a fixed constant and a variable data vector. It uses precomputed coefficients, which are stored in ROMs for computing the matrix vector products in DCT. It uses lookup tables and adders instead of multipliers [19]. Most of the DA based DCT techniques use the conventional DCT algorithm along with some memory reduction techniques. In this procedure, partial products of the DCT are already computed and stored in ROM. These saved partial products are accessed by the address and accumulated for producing the result of the multiplication. The major overheads of DA based implementations are the size of the ROMs and the access time of the ROMs. Unfortunately, the size of the memory increases exponentially when the number of inputs and precision increase.

### 2.3. New Distributed Arithmetic

NEDA is one of the popular designs of DA based DCT architecture [11]. It is multiplier-less as well as a ROM-less optimized implementation. This architecture decreases the complexity up to some extent by using CSD and sharing of common subexpression. This approach leads to produce minimal shift-add expressions for DCT implementation. Due to this optimization, low power and high throughput DCT architecture is achieved. However, with these advantages, NEDA has some drawbacks as well [29]. The major disadvantage is due to parallel data input screening, which leads to restricting the operating frequency.

### 2.4. Coordinate Rotation Digital Computer

CORDIC introduced a cost efficient technique for DCT computation. The CORDIC scheme uses dynamic transformation, which leads to high-power consumption. In [21], the authors presented CORDIC algorithm based DCT architecture, by using Loeffler algorithm, and facing same disadvantage of high-power dissipation.

Other than these approaches, some joint optimization techniques are used to reduce the complexity of the DCT architecture, which results in reducing the power dissipation of the system. Some of them use signal correlation property to design low power architecture [30]. In [31], the authors optimized the design by using Huffman tables, quantization, and DCT. Hsu and Cheng in [32] investigated prediction algorithm to reduce the resource usage of the DCT architecture. In [33], the authors claimed least hardware resource usage with efficient power consumption. Their architecture is based on joint optimization of CSD, CSE, and quantization.

In this paper, a novel multiplier-less DCT architecture is proposed to save the hardware resources in terms of adders/subtractors and the number of slices used. The proposed design also meets real time DCT requirements of various coding standards, such as H.261, H.263, MPEG1, MPEG2, and MPEG4, when operating at different frequencies with low dynamic power consumption.

### 2.5. Proposed Architecture for DCT

The proposed architecture is optimized by CSD and sharing of common subexpressions between all DCT coefficients. This design consists of 5-stage pipelined architecture, which increases the throughput of the system. In this section, brief descriptions of DCT coefficient symmetry, CSD, and CSE are discussed with the proposed architecture.

#### 2.5.1. DCT Coefficients Symmetry

It is noticed that 8 × 8 block DCT coefficient matrix has symmetry between its rows and columns. In each column, all 7 coefficients from *c*_1_ to *c*_7_ are presented once, except *c*_4_ which comes twice, either in positive value or in negative value. *c*_4_ is always in 1st and 5th rows. Odd coefficients (*c*_1_, *c*_3_, *c*_5_, and *c*_7_) are placed in 2nd, 4th, 6th, and 8th rows. Remaining even coefficients (*c*_2_ and *c*_6_) are sharing 3rd and 7th rows. These characteristics of the DCT matrix allowed making hardware of just one multiplication module for the respective coefficient and then reusing it to complete the computation of DCT result. DCT coefficient matrix “*D*” is illustrated in

(3) $$\begin{array}{c} D=\left[\begin{array}{cccccccc} c_{4} & c_{4} & c_{4} & c_{4} & c_{4} & c_{4} & c_{4} & c_{4}\\ c_{1} & c_{3} & c_{5} & c_{7} & -c_{7} & -c_{5} & -c_{3} & -c_{1}\\ c_{2} & c_{6} & -c_{6} & -c_{2} & -c_{2} & -c_{6} & c_{6} & c_{2}\\ c_{3} & -c_{7} & -c_{1} & -c_{5} & c_{5} & c_{1} & c_{7} & -c_{3}\\ c_{4} & -c_{4} & -c_{4} & c_{4} & c_{4} & -c_{4} & -c_{4} & c_{4}\\ c_{5} & -c_{1} & c_{7} & c_{3} & -c_{3} & -c_{7} & c_{1} & -c_{5}\\ c_{6} & -c_{2} & c_{2} & -c_{6} & -c_{6} & c_{2} & -c_{2} & c_{6}\\ c_{7} & -c_{5} & c_{3} & -c_{1} & c_{1} & -c_{3} & c_{5} & -c_{7} \end{array}\right]. \end{array}$$

**Figure 1:**
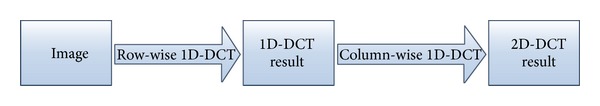
2D-DCT using separability property.

The DCT separability property allows computing 2D-DCT of the image in two steps by successive 1D-DCT operations on row and columns of the image, which leads to improve the speed of the system. This property is also applicable for inverse DCT as well [34]. This idea is graphically illustrated as in Figure 1.

Mathematically, in matrix form, this property can be represented as

(4) $$\begin{array}{c} 1\text{D-DCT}=D\times I\\ 2\text{D-DCT}=\left(1\text{D-DCT}\right)\times D^{T}. \end{array}$$

#### 2.5.2. Canonical Signed Digit Representation

CSD representation is normally used to minimize the number of additions and shift operations in each fixed coefficient multiplication on the cost of subtraction operation. It presents the number with the minimal nonzero digits occurrences for a constant. The CSD format can decrease 33% of nonzero digits compared to the binary format [35].

The proposed scheme especially incorporates the CSD method for more efficient hardware usage and reduces the hardware complexity significantly in multiplier-less implementation of DCT. CSD form notation is

(5) $$\begin{array}{c} s=\overset{k-1}{\underset{i=0}{\sum }}a_{i}2^{-i}, \end{array}$$

where *a*_*i*_ is in the set {−1,0, 1} for each *i*.

According to IEEE 1180–1990 [36], 12-bit precision is used in order to confirm the accuracy specifications of the DCT, so the fixed-point implementation is quite acceptable. The DCT coefficients in fixed point CSD format with 12-bit precision and reduction of nonzero bits for each coefficient are shown in Table 2 (in Table 2, $\overset{-}{1}$ represents −1).

#### 2.5.3. Efficient Usage of CSE for DCT Implementation

The fixed coefficients of a DCT in CSD format have some common subexpressions. Common subexpression means that some of the bit patterns occur more than once in any expression. Close observations on the fixed coefficients extract some common subexpressions, which can be easily eliminated [37].

This work proposes a new CSE approach for DCT architecture, which is not only used for fixed coefficients of a DCT in CSD but also shares the expressions with intermediate DCT coefficients results as well. Implementation of common subexpression sharing with the characteristics of the DCT reduces the number of resources (number of adders or/and subtractors), which results in low power and area efficient design. Detailed common subexpression sharing is shown below:

(6) c1=0.100001−01−︸−B000c2=0.10001−01−︸−B001︷E00c3=0.101−︸A0101︸B0101−︸A0c4=0.101−︸A01−0101︸B000c5=0.01001︸D001−0001︸−Cc6=0.0101−︸A00010001−︸Cc7=0.00101−︸A0010001−︸C.

Common subexpression terms are

(7) $$\begin{array}{c} \text{A}=\text{input}\gg 1-\text{input}\gg 3\\ \text{B}=\text{input}\gg 5+\text{input}\gg 7\\ \text{C}=\text{input}\gg 8-\text{input}\gg 12. \end{array}$$

Noncommon terms are

(8) $$\begin{array}{c} \text{D}=\text{input}\gg 2+\text{input}\gg 5\\ \text{E}=\text{input}\gg 1+\text{input}\gg 10. \end{array}$$

Symbol “≫*n*” represents the right shift operation by *n-bit. *The common subexpression term *A* has the highest priority and is computed in the first stage because it is used two times in one coefficient. The remaining terms (*B* to *E*) are computed in the second stage, which reduces the pipeline register width of the first stage. This reduction decreases the power consumption and silicon area with keeping the high operating frequency.

**Figure 2:**
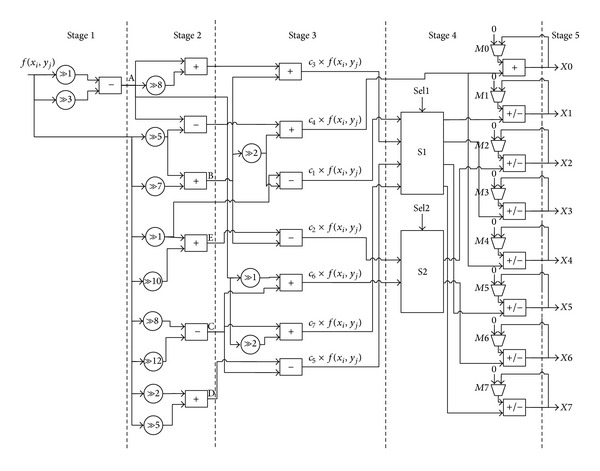
Proposed 5-stage pipelined DCT architecture.

Figure 2 shows the proposed five-stage pipelined DCT architecture based on novel CSE optimization. Term *A* is computed in the first stage of the pipelined architecture. The remaining terms are generated in the second stage. The fixed shifters are used in this hardware design instead of barrel shifters. Fixed shifting is easily implemented by manipulation of the hard-wires and it consumes low power. The bit width of the data-path design is different at different stages, which provide efficient use of the area and reducing of the power consumption.

In stage 3, all products of the input and the coefficients are computed. Moreover, these partial product results are forwarded to the respective adders in the 4th stage. There are two selectors S1 and S2 in the 4th stage, which are used to decide the destination of the results. S1 takes the multiplication result of input pixel *f*(*x*_*i*_, *y*_*i*_) by *c*_1_, *c*_3_, *c*_5_, and *c*_7_, while S2 takes the multiple calculation of *c*_2_ and *c*_6_. This selection is based upon the symmetry of the DCT coefficient matrix. The selection of S1 and S2 is defined in Tables 3 and 4, respectively.

The 4th-stage add/sub is adding or subtracting respective selection outputs with the previous partial product result. The selection of add/sub is according to the magnitude of the respective DCT coefficient. M0 to M7 multiplexers select that the addition/subtraction is with the feedback value (DCT coefficient has positive sign or negative sign) or with zero (when reset and new block of the DCT is going to compute). Finally, 5th-stage provides the eight parallel outputs of the result.

## 3. Results and Discussion

To emphasize the CSE sharing with this approach for computing DCT results, a Hardware Descriptive Language (HDL), Verilog model is designed and compared with other recent literature techniques. The comprehensive comparisons are examined under the same platforms to validate the results. The proposed architecture is synthesized using Xilinx ISE 10.1 software for Xilinx FPGAs (Spartan-3 and Virtex-II) and Quartus II 13.0 tool for Altera FPGA (Cyclone II). The information related to the number of resources used, the maximum operating frequency, and the number of slices (required area) used by the proposed architecture is depicted after performing the post place and route procedure. The power consumption of the proposed architecture is estimated by the XPower tool of Xilinx for Xilinx FPGAs. However, the PowerPlay tool of Altera is used for Altera FPGA to determine the power dissipation of the proposed system.  Table 5 shows the description of the platforms used for the experiments. The detailed information about family, device, and speed grade, of Xilinx and Altera FPGAs, is illustrated.  Table 5 also provides the details of power analysis of the proposed architecture. These details include static power, dynamic power, maximum operating frequency, and design voltage of the design. The power analysis is according to the design voltage and clock frequency.

Table 6 shows the resource usages of the proposed design in terms of adders, subtractors, add/sub, selectors, and the number of fixed shift counts. The proposed architecture uses only 22 adders/subtractors in total with no multiplier for computing the DCT results. Furthermore, the proposed design does not use any Digital Signal Processing (DSP) slices and memory modules to implement the design.

1D-DCT computation requires 8 clock cycles. However, initial pipeline filling cost of 4 clock cycles increases the first 1D-DCT computation clock cycles to 12. The total number of clock cycles required for 2D-DCT is 76 clock cycles, which is comprised of 4 cycles for pipeline filling, 8 clock cycles for the first 1D-DCT computation, and 64 clock cycles for computing 8 × 8 2D-DCT. Remaining seven 1D-DCT results are calculated in parallel with 2D-DCT computation; therefore, there will be no effect in total clock cycles.

Table 7 illustrates the comparison of the minimum number of resource usage in terms of adders/subtractors. The direct realization of DA-based DCT implementation requires 308 adders. In [11], the authors reduce the number of adders to 84. Optimizations based on CSD designs [26, 38] consume 69 and 67 adders, respectively. The scheme proposed in [33] based on CSD-CSE joint optimization uses 72 adders to compute DCT. It can be observed that Zhenwei et al. [41] require 26 adders while the proposed design involves only 22 adders for DCT implementation.

Moreover, the performance analysis of the different 1D-DCT multiplier-less architectures is compared with the proposed design in Tables 8 and 9. The comparison using Xilinx FPGAs shows that the proposed method uses the least slices for 1D-DCT than the recent conventional multiplier-less architectures. The slices occupied in [40], 936 and 793, are the worst cases while using Virtex-II and Spartan-3, respectively. Furthermore, the comparison using Altera FPGA also proves that the suggested architecture has consumed less logic elements than the other recent methods. In [38], the authors presented the DCT architecture with 1146 logic elements. However, the proposed design utilizes only 713 logic elements.

Furthermore, 12-bit precision is used for achieving more precise DCT results. However, in [40], the authors used 9 bits for precision and achieving high PSNR (peak-signal-to-noise-ratio), but according to IEEE standards [36], 12-bit fixed constant precision is best trade-off for DCT implementation. The proposed design not only fulfills the IEEE criteria of 12-bit precision of DCT constant coefficient but also uses the variable-bit data width in intermediate links of the architecture. The 8-bit input is fed to the system. On the first stage of the pipelined architecture, only *A* term is computed and its data bus width is increased to 11 bits. Furthermore, at the second stage, the resultant terms (*B* to *E*) have different bus widths according to their results. This technique leads to reduce the power dissipation and silicon area of the design.

**Figure 3:**
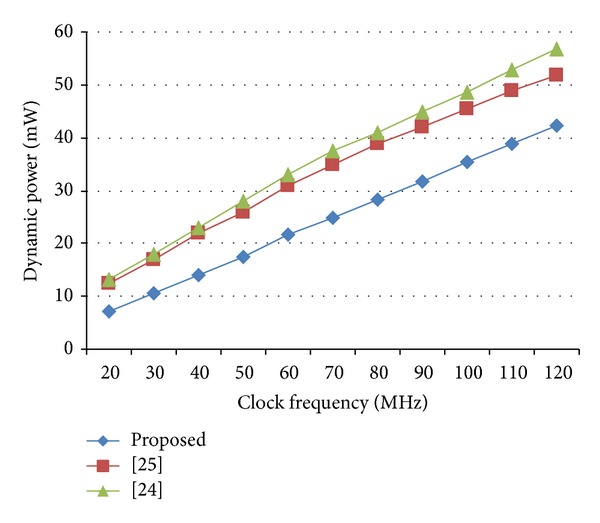
Dynamic power consumption estimation per sample with 1.2 V design.

The most remarkable feature of the proposed design is its low power consumption. Tables 8 and 9 also reveal that the proposed architecture has the least power dissipation in each case than the other implementations. It consumes only 23 mW for computing DCT results on low cost Spartan family. However, DA-based architecture of Chen et al. [40] has consumed 45 mW using the same device. Furthermore, the recommended architecture has significant results in terms of power dissipation when implemented on Virtex family FPGAs too. It draws only 35 mW, which is lower than the other implementations tested on the same platform. Modified Loeffler based implementations [26, 38], designed on Altera FPGA, consume 57 mW and 52 mW, respectively. On the other hand, using the same platform, the proposed design merely dissipates 42 mW.  Figure 3 shows the comparison between the proposed architecture and some recent fast DCT implementations, operating at different frequencies.

In [26, 33, 38, 40], all methods need 8 inputs at a time, which introduces the multiread port memory in their architectures at the input stage. However, the proposed design needs single port memory for feeding the inputs to the system. Multiread port memory consumes more power as well as area than the single port memory. The area is increased more than two times proportional to the number of ports [42]. This approach reduces the silicon area usage and decreases the power consumption of the system.

The proposed architecture also achieves 163.84 MHz, 205 MHz, and 191.79 MHz, maximum operating frequency on Spartan-3, Virtex-II, and Cyclone II, respectively. These results are quite remarkable and easily fulfil the throughput criteria of standard image and video coders, keeping the power consumption as low as possible. However, Table 10 shows the applications to various image and video standards. For larger picture size and higher frame rate, the proposed design can be simply used with higher operating clock frequencies to achieve the real time constraints. Furthermore, it also correlates the dynamic power of the proposed architecture, operating at different frequencies. The Spartan-3 platform is used to achieve the results of Table 10. These results prove that the proposed design throughput easily meets the real time encoding requirements.

### 3.1. Image Results

**Figure 4:**
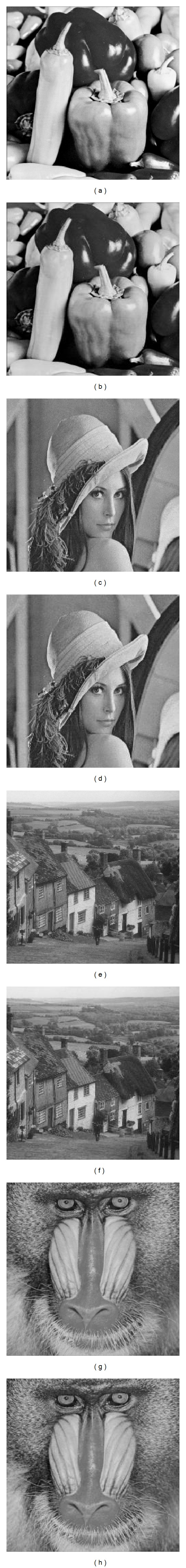
Original standard testing images and their reconstructed images. (a) Original Image “Peppers” (b) Reconstructed Image “Peppers” (PSNR = 52.94 dB) (c) Original Image “Lena” (d) Reconstructed Image “Lena” (PSNR = 54.04 dB) (e) Original Image “Goldhill” (f) Reconstructed Image “Goldhill” (PSNR = 54.64 dB) (g) Original Image “Mandrill” (h) Reconstructed Image “Mandrill” (PSNR = 53.82 dB).

Standard images “Peppers,” “Lena,” “Goldhill,” and “Mandrill” are used to simulate the proposed design efficiency. These images are composed of 256 × 256 pixels, with each pixel being represented by 8 bits corresponding to 256 gray levels. To examine the quality of the reconstructed images using an FPGA (Spartan-3) prototype of the proposed architecture, an image is saved in a ROM to avoid the transmission time between the PC and FPGA. The proposed DCT architecture takes the input pixels one by one from the memory and generates the 2D-DCT result on the output port. The transformed output result is fed into MATLAB (Version: R2013a) tool (inverse 2D-DCT function) to reconstruct the image. Then PSNR values are computed in MATLAB by using “peakpsnr” function of MATLAB. This function uses the reconstructed image and original image as a reference to calculate the PSNR value. The proposed design achieves significant PSNR values, which are close to 54.64 dB. However, recent modern techniques [33, 40] are able to achieve PSNR values maximally 33.24 dB and 47 dB, respectively.  Figure 4 shows the original test images and the reconstructed images computed by the proposed 2D-DCT model.

**Figure 5:**
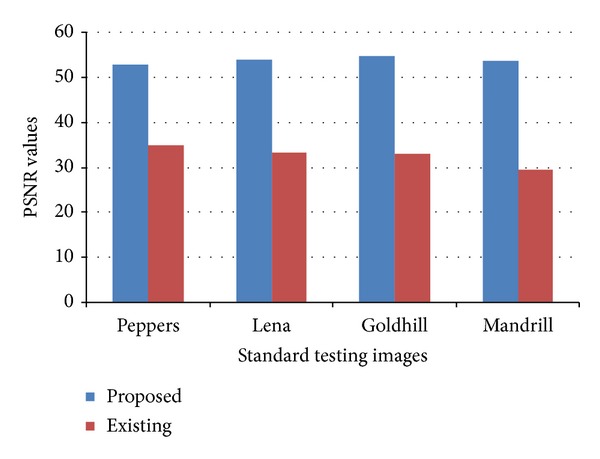
PSNR analysis on different standard testing images.

PSNR of different standard gray level test images are evaluated and compared.  Figure 5 illustrates the comparison analysis.

From the above discussed results and comparisons, it is clear that the proposed system has significantly high efficiency among all state-of-the-art literature works. The results are examined on different devices to enhance the comparison. Moreover, it can be operated at high frequencies and it consumes lower power. Furthermore, this architecture occupies a less silicon area by reducing the number of adders used.

## 4. Conclusion

In this study, a high-speed, low power, and area efficient multiplier-less DCT architecture is proposed for DCT based image compression. This research presents a novel method for the intermediate computation results of the DCT algorithm based on the CSD and CSE. This efficient system gives promising PSNR value in reconstructing the compressed image. According to the experimental results, the proposed approach yielded better performance in terms of the minimum number of adders used to compute the DCT, when compared to other popular methods available in the recent literatures. Furthermore, the results stated that the proposed method consumes less power than the other published approaches. To authenticate the credibility of the results, the proposed design is tested on different platforms. Moreover, this architecture can be easily equipped with the telemetry imaging application, any portable devices or mobile applications. It can be very effectively applied in H.261, H.263, H.264, MPEG-1, MPEG2, MPEG-4 video coding standard schemes for internet video streaming, video conferencing, and many other high density TV applications.

In future, this work can be employed for different versions of transforms, such as discrete wavelet transform or lift-up wavelet transform. Systolic architecture aspect of the proposed design could be also explored, which would focus to increase the throughput. The computation time could be decreased using the advanced parallel processing techniques. The extension of the developed scheme, to processing the real time video, is also a challenging issue of future research. Larger block size DCT, like 64 × 64 2D-DCT, is also one branch for the researchers to investigate with this approach.

## Figures and Tables

**Table 1:** Number of arithmetic operations for 8-point DCT computation of some well-known algorithms.

Algorithm	Additions	Multiplications
Loeffler et al. [24]	29	11
Suehiro and Hatori [27]	29	12
Lee [23]	29	12
Wang [28]	29	13
Chen et al. [22]	26	16

**Table 2:** 12-bit precise DCT coefficient in CSD form and reduction of nonzero bits.

Coefficient	Decimal value	Binary representation	CSD representation	Reduction of Nonzero bits
*c*_1_	0.4904	0.011111011000	$0.00000\overset{-}{1}0\overset{-}{1}0001$	4
*c*_2_	0.4619	0.011101100100	$0.1000\overset{-}{1}0\overset{-}{1}00100$	2
*c*_3_	0.4157	0.011010100110	$0.10\overset{-}{1}0101010\overset{-}{1}0$	0
*c*_4_	0.3536	0.010110101000	$0.10\overset{-}{1}0\overset{-}{1}0101000$	0
*c*_5_	0.2778	0.010001110001	$0.0100100\overset{-}{1}0001$	1
*c*_6_	0.1913	0.001100001111	$0.010\overset{-}{1}0001000\overset{-}{1}$	2
*c*_7_	0.0975	0.000110001111	$0.0010\overset{-}{1}001000\overset{-}{1}$	2

**Table 3:** Selector S1 attributes according to selection Sel1.

Sel1	Operation			
	Output 1	Output 2	Output 3	Output 4
00	*c*_1_ × *f*(*x*_*i*_, *y*_*j*_)	*c*_3_ × *f*(*x*_*i*_, *y*_*j*_)	*c*_5_ × *f*(*x*_*i*_, *y*_*j*_)	*c*_7_ × *f*(*x*_*i*_, *y*_*j*_)
01	*c*_3_ × *f*(*x*_*i*_, *y*_*j*_)	*c*_7_ × *f*(*x*_*i*_, *y*_*j*_)	*c*_1_ × *f*(*x*_*i*_, *y*_*j*_)	*c*_5_ × *f*(*x*_*i*_, *y*_*j*_)
10	*c*_5_ × *f*(*x*_*i*_, *y*_*j*_)	*c*_1_ × *f*(*x*_*i*_, *y*_*j*_)	*c*_7_ × *f*(*x*_*i*_, *y*_*j*_)	*c*_3_ × *f*(*x*_*i*_, *y*_*j*_)
11	*c*_7_ × *f*(*x*_*i*_, *y*_*j*_)	*c*_5_ × *f*(*x*_*i*_, *y*_*j*_)	*c*_3_ × *f*(*x*_*i*_, *y*_*j*_)	*c*_1_ × *f*(*x*_*i*_, *y*_*j*_)

**Table 4:** Selector S2 attributes according to selection Sel2.

Sel2	Operation	
	Output 1	Output 2
0	*c*_2_ × *f*(*x*_*i*_, *y*_*j*_)	*c*_6_ × *f*(*x*_*i*_, *y*_*j*_)
1	*c*_6_ × *f*(*x*_*i*_, *y*_*j*_)	*c*_2_ × *f*(*x*_*i*_, *y*_*j*_)

**Table 5:** Description of the platforms used for the experiments with power analysis.

	Xilinx FPGAs			Altera FPGAs
Family	Virtex-II	Virtex-II	Spartan-3	Cyclone II
Device	XC2VP30	XC2VP50	XC2S200	EP2C35
Speed grade	−5	−5	−5	6
Design voltage (V)	1.4	1.4	1.2	1.2
Max. clock frequency (MHz)	205	205	163.84	191.79
Static power (mW)	768	768	44	83
Dynamic power (mW)	66	66	23	42

**Table 6:** Resource usages and DCT computing cycles of the proposed architecture.

Total number of adders	9
Total number of subtractors	6
Total number of add/sub	7
Total number of fixed shifts	13
Total number of selectors	2
DSP slices	0
Memory modules	0
Total number of clock cycles for computing 1D-DCT	4 + 8
Total number of clock cycles for computing 8 × 8 2D-DCT	12 + 64

**Table 7:** Macrostatistics of 1D-DCT implementation.

Method	[11]	[33]	[38]	[26]	[39]	[40]	[41]	Proposed
Adders	84	72	69	67	56	31	26	**22**

**Table 8:** Performance analysis of different 1D-DCT architectures on Xilinx FPGAs.

FPGA chip	XC2VP30		XC2VP50		XC3S200	
Architecture	[40]	Proposed	[33]	Proposed	[40]	Proposed
Implementation	DA	CSD + New-CSE	CSD + CSE	CSD + New-CSE	DA	CSD + New-CSE
Precision (bits)	9	12	11	12	9	12
Number of slices	936	347	454	347	793	340
Operating clock frequency (MHz)	99	205	119	120	61	163.84
Dynamic power dissipation (mW)	83.4	66	39	35	45	23
Multiport input memory (number of read ports)	Yes (8)	No (1)	Yes (8)	No (1)	Yes (8)	No (1)

**Table 9:** Performance analysis of different 1D-DCT architectures on Altera FPGA.

FPGA chip	Cyclone II (EP2C35F672C6)		
Architecture	[38]	[26]	Proposed
Implementation	Modified Loeffler	Modified Loeffler	CSD + New-CSE
Precision (bits)	12	12	12
Logic elements	1146	1109	713
Operating clock frequency (MHz)	128.25	139.55	191.79
Dynamic power dissipation (mW)	57	52	42
Multiport input memory (number of read ports)	Yes (8)	Yes (8)	No (1)

**Table 10:** Proposed design applications to various image/video standards (8 × 8 block size).

Applications	Data rate *H* × *V* × *f*	Operating frequency (MHz)	Number of frames computed	Dynamic power consumption (mW)
JPEG	640 × 480	0.38	1	0.01
H.263-QCIF	176 × 144 × 10	0.26	10	0.01
H.263-CIF	352 × 288 × 15	1.52	15	0.07
MPEG-1	352 × 240 × 30	2.54	30	0.11
MPEG-2	720 × 480 × 30	10.37	30	0.45
MPEG-2 (PAL)	720 × 576 × 25	10.37	25	0.45
MPEG-2 (HD1)	1440 × 1080 × 30	46.66	30	2.04
MPEG-2 (HD2)	1920 × 1080 × 30	62.21	30	2.73
